# fAFLP analysis of Brazilian *Bacillus thuringiensis* isolates

**DOI:** 10.1186/2193-1801-3-256

**Published:** 2014-05-20

**Authors:** Fernando Hercos Valicente, Rosane Bezerra da Silva

**Affiliations:** Embrapa Milho e Sorgo, Rod. MG 424 KM 65, CEP 35701-970 Sete Lagoas, MG Brazil; Department of Biotechnology, Campus Universitário, CEP 37200-000 Lavras, MG Brazil

**Keywords:** AFLP, Genetic diversity, *Bacillus thuringiensis*, Restriction enzymes

## Abstract

A total of 65 *Bacillus thuringiensis* (Bt) isolates were subjected to analysis of genetic relationship using fAFLP (fluorescent Fragment Length Polymorphism), in order to determine the genetic diversity within a group of Bt strains. 26 strains from different subspecies were identified as it follows: 9 kindly provided by the USDA (United States Department of Agriculture), 9 kindly provided by the Institute Pasteur and eight from Embrapa Maize and Sorghum Bt Collection, and 39 strains with no subspecies information also from Embrapa’s Bt Collection. DNA sample was double digested with restriction enzymes *Eco*RI and *Mse*I, and the fragments were linked to adapters. Selective amplification reactions were performed using five primer combinations and the amplified fragments were separated by gel electrophoresis on an ABI377 sequencer. Genetic distances were obtained by the complement of the Jaccard coefficient and the groups were performed by the UPGMA method. Five primer combinations generated 495 scorable fragments and 483 were found to be polymorphic. Out of 26 subspecies, strains 344 and T09 (*B. thuringiensis* subsp. *tolworthi*) showed the highest similarity (15%), while isolates HD3 *B. thuringiensis* subsp *finitimus* and T24 *B. thuringiensis* subsp *neoleonensis* were the most genetically distant (92%). *B. thuringiensis* isolates with no subspecies identification, found in samples from Goiás State showed higher similarity forming a group with an average distance of 6%, and the closest subspecies to this group was *B. thuringiensis* subsp *thuringiensis* (HD2) with 52% of similarity. This similarity may be due to the fact that these organism exchange genetic material by conjugation, and it is relatively common to have evolutionary characteristics of their ancestors.

## Background

*Bacillus thuringiensis* (Bt) is a rod-shaped, Gram-positive soil bacterium characterized by its ability to produce crystalline inclusions called “Cry proteins, Cry toxins or Bt toxins” during sporulation. The crystalline inclusions along with the spores have a high potential to control a great number of insect pests belonging to the order Lepidoptera, Diptera and Coleoptera (Vidyarthi et al. 2002; Yamamoto and Dean 2000). *B. thuringiensis* can be found in different substrates such as soil, water, plant surfaces, dead insects, grain dust, and stored grain (Glare and O’Callaghan 2000; Valicente and Barreto 2003), and it is a useful alternative or zment to synthetic chemical pesticide applications in commercial agriculture, horticulture, forest management, and mosquito control (De Maagd et al. 1999). *B. thuringiensis* also has high genetic variability and are widely distributed in nature (Vilas-Boas et al. 2002 Martin and Travers 1989 Hossain et al. 1997). The polymerase chain reaction (PCR) has been used to identify specific *cry* genes and characterize *B. thuringiensis* strains with a high degree of success (Chak et al. 1994; Cerón et al. 1994, 1995; Shangkuan et al. 2001; Lima et al. 2002; Valicente et al., 2010).

The Amplified Fragment Length Polymorphism (AFLP) technique is based on the detection of genomic restriction fragments by PCR amplification, and can be used for DNAs of any origin or complexity (Vos et al. 1995). AFLP may be used as a tool for bacterial taxonomy and has shown utility in detecting molecular variability in very closely related bacterial strains (Jansen et al. 1997; Burke et al. 2004; Grady et al. 2001). The fAFLP (flurescent Amplified Fragment Length Polymorphism) use markers with fluorescent substances, the use of primers labeled with a fluorochrome, associated with automatic sequencers and a large capacity for computational analysis (Ryu et al. 2005; Burke et al. 2004).

*Spodoptera frugiperda* (Smith) is responsible for significant losses in maize production in Brazil. Its control is mainly achieved using chemical insecticides. However, biological control using *B. thuringiensis* and the production of genetically modified plants with insect resistance genes are excellent alternatives to control this insect (Valicente and Barreto 2003).

The purpose of the present study was to use fAFLP for molecular analysis and characterization of *B. thuringiensis* strains that were previously tested against *S. frugiperd*a, and to determine the degree of genetic variability and correlation between different genotypes of public strains of *B. thuringiensis* and strains sampled in different Brazilian regions.

## Materials and methods

### *B. thuringiensis* strains

Of a total of 65 *B. thuringiensis* strains used in this research (Table 1), 26 strains from different subspecies were identified as it follows: 9 kindly provided by the USDA (United States Department of Agriculture), 9 kindly provided by the Institute Pasteur and eight from Embrapa Maize and Sorghum Bt Collection. Also, 39 strains with no subspecies information also from Embrapa’s Bt Collection (Table 1). The words subspecies and serovar were used interchangeably as given by Zeigler (1999). All strains have been previously tested against fall armyworm, *S. frugiperda* (Lepidoptera: Noctuidae J.E. Smith) (Valicente and Fonseca 2004; Valicente and Barreto 2003), and showed different toxicity activity. These strains are stored in glycerol in a freezer at – 20°C.

**Table 1:** ***Bacillus thuringiensis*** **serovars used in this study, with mortality against fall armyworm,** ***Spodoptera frugiperda***

N°	Bt subspecies	Strain identification	Mortality (%)*	Origin	N°	Strain identification	Mortality (%)*	Origin
**1**	*alesti*	HD-4	6.8	USA	**34**	1109 N	100	Goiás State/Brazil
**2**	*alesti*	348B	100	Paraná State/Brazil	**35**	1145B	100	Goiás State/Brazil
**3**	*aizawai*	T-07	80.8	France	**36**	1145C	100	Goiás State/Brazil
**4**	*aizawai*	HD-11	7.8	USA	**37**	1148 F	100	Goiás State/Brazil
**5**	*galleriae*	462A	100	Paraná State/Brazil	**38**	1132E	100	Goiás State/Brazil
**6**	*galleriae*	474	100	Paraná State/Brazil	**39**	1135B	100	Goiás State/Brazil
**7**	*galleriae*	348 L	100	Paraná State/Brazil	**40**	1136B	100	Goiás State/Brazil
**8**	*galleriare*	HD-29	12.8	USA	**41**	1139 K	100	Goiás State/Brazil
**9**	*tolworthi*	344	100	Paraná State/Brazil	**42**	BTLM	100	Goiás State/Brazil
**10**	*tolworthi*	T-09	100	Françe	**43**	1644	100	Paraná State/Brazil
**11**	*tolworthi*	426	100	Ceará State/Brazil	**44**	1641	100	Paraná State/Brazil
**12**	*tolworthi*	461A	100	Paraná State/Brazil	**45**	701A	100	São Paulo State/Brazil
**13**	*darmstadiensis*	460	100	Paraná State/Brazil	**46**	1658	100	São Paulo State/Brazil
**14**	*darmstadiensis*	T-10	77.9	France	**47**	1646	100	São Paulo State/Brazil
**15**	*entomocidus*	T-06	9.8	France	**48**	701B	100	São Paulo State/Brazil
**16**	*neoleonensis*	T-24	17.9	France	**49**	1648	100	São Paulo State/Brazil
**17**	*mexicanensis*	T-27	17	France	**50**	1438	100	Sergipe State/Brazil
**18**	*japonensis*	T-23	33.5	France	**51**	1438H	100	Sergipe State/Brazil
**19**	*indiana*	T-16	12.2	France	**52**	1089E	95.8	Minas Gerais/State/Brazil
**20**	*kurstaki*	HD-73	2.7	USA	**53**	1091H	95.8	Minas Gerais State/Brazil
**21**	*kurstaki*	HD-1	0	USA	**54**	1604D	95.2	Amazônia State/Brazil
**22**	*morrisoni*	HD-12	28	USA	**55**	1605	95.2	Amazônia State/Brazil
**23**	*dendrolimus*	HD-7	5.4	USA	**56**	1657	100	Amazônia State/Brazil
**24**	*finitimus*	HD-3	5.2	USA	**57**	1656	100	Alagoas State/Brazil
**25**	*thuringiensis*	HD-2	37.8	USA	**58**	1603B	100	Santa Catarina State/Brazil
**26**	*israelensis*	T-14	0	France	**59**	1626C	97.6	Maranhão State/Brazil
**27**		1119C	100	Goiás State/Brazil	**60**	702	97.6	Mato Grosso State/Brazil
**28**		1124E	100	Goiás State/Brazil	**61**	1354	100	Minas Gerais State/Brazil
**29**		1131^A^	100	Goiás State/Brazil	**62**	1355SLO	100	Minas Gerais State/Brazil
**30**		1131C	100	Goiás State/Brazil	**63**	1357E	100	Paraná State/Brazil
**31**		1132^A^	100	Goiás State/Brazil	**64**	566	100	Paraná State/Brazil
**32**		1132C	100	Goiás State/Brazil	**65**	1355LM	100	Minas Gerais State/Brazil
**33**		1138G	100	Goiás State/Brazil				

*Mortality results from Valicente and Barreto 2003 and Valicente and Fonseca 2004.

### Bacterial cultures

*B. thuringiensis* isolates were inoculated into 5 ml LB medium enriched with salts and glucose (all expressed in g.l^−1^, 0.002 g of FeSO4, 0.02 g of ZnSO4, 0.02 g of MnSO4, 0.3 g of MgSO4) and 2% glucose, with pH adjusted to 7.2. The shaking flasks were incubated at a stirrer speed of 250 rpm for 16 h at 28°C. Colonies were observed on a phase contrast microscope.

### DNA extraction

Genomic DNA from *B. thuringiensis* strains was isolated and purified according to Shuhaimi et al. (2001) with modifications. A volume of 5 ml of an overnight culture was centrifuged at 4600 g for 5 min. The pellet was washed with sterile distilled water and resuspended in 700 μl glucose/Tris/EDTA buffer (50 mM glucose, 25 mM Tris/HCl and 10 mM EDTA, pH 8). Lysozyme was added to the bacterial suspension at 20 mg/ml and incubated at 37°C for 30 min. The cells were additionally lysed by adding 15 μl 20% (w/v) SDS. Five μl of proteinase K at 20 mg/ml was added and incubated at 65°C for 1 h to complete protein digestion. Five hundred μl of Phenol/chloroform/isoamyl alcohol (25:24:1 by vol.) was added and centrifuged at 12.000 g for 1 min. Three hundred μl of the aqueous upper layer was then transferred into a sterile new tube and an equal volume of 3 M potassium acetate (pH 5.2), and a double volume of 2-propanol were added. The mixture was then centrifuged at 12.000 g for 7 min. The supernatant was carefully discarded. The pellet was washed with 70% (v/v) ethanol by centrifuging at 12.000 g for 5 min. The ethanol was discarded and the pellet containing the chromosomal DNA resuspended in 150 μl of Tris-EDTA pH 8.0 (10 mM Tris–HCl, 1 mM EDTA) and stored at −20°C until further use.

### fAFLP analysis of DNA samples

The fAFLP reactions were performed using to the Applied Biosystems AFLP™ microbial fingerprinting Kit with the genomic DNA (500 ng) being digested with 5U *Eco*RI and 2,5 U of *Mse*I (Invitrogen®, Carlsbad, CA, EUA) in 1,25 μl of buffer 10× with final volume of 10 uL. After the digestions, were added 1 μl of *Eco*RI and *Mse*I adapters (2 mM), 1.5 U of T4 DNA ligase buffer with T4 DNA ligase of (50 mM Tris–HCl, pH 7.8, 10 mM MgCl^2^, 10 mM dithiothreitol, 1 mM ATP, 25 g/ml bovine serum albumin). The binding reactions were conducted at 20°C for 2 h, and amplicons produced were dissolved in 30 μl of TE buffer (10 mM Tris–HCl, 1 mM EDTA, pH 8.0) and used as template in pre-selective amplification reactions. The binding sites of pre-selective primers are the sequences of the adapters of restriction sites that provide a selection of restriction fragments obtained. The reactions were obtained using a 2 μl of diluted DNA, 7.5 μl of Core Mix and 0.5 μl of *Eco*RI primers (1 μM) and *Mse*I (5 mM) were considered as pre-selective. Amplification conditions were: 72°C for 2 minutes, 20 cycles starting at 94°C for 20 s, 56°C for 30 s and 72°C for 2 minutes. Products amplification were electrophoresed on 0.8% agarose gels and subsequently stained colored with ethidium bromide (0.5 mg/ml), and observations about the presence of bands at most of 500 bp. The pre-amplified products were diluted (1:15) with TE buffer and 1.5 μl were used in the pre-amplification reaction along with 7.5 μl of buffer, nucleotides and AmpliTaq® DNA polymerase, a mixture called “Core Mix, 0.5 μl of the *Mse*I primer (5 μM) and 0.5 μl of *Eco*RI primer, labeled with fluorescence (1 μM). Selective amplifications were performed in 20 μl reactions with the following steps: 94°C for 30 s, 65°C for 30 s, and 72°C for 1 min for 1 cycle and then lowering the annealing temperature by 1°C each cycle to 56°C, followed by an additional 20 cycles at a 56°C. Five primer combinations were used in the selective amplification (*Eco*RI -AC + *Mse*I -CG; *Eco*RI -G + *Mse*I -CC; *Eco*RI -C + *Mse*I -G; *Eco*RI −0 + *Mse*I -CG; *Eco*RI -C + *Mse*I -CG).

### Sequencing eletrophoresis of polyacrylamide gel and data analysis

Each sample was loaded onto a 5% Long-Ranger denaturing gel which was run in an ABI PRISMTM 377 DNA Sequencer (PE-Applied Biosystems) for 4 h at 2,500 V. The electrophoresis images obtained from each reaction were analyzed using the GENESCAN program version 3.1 (Applied Biosystems). The Genotyper program version 2.5 (Applied Biosystems) was used to check the presence (coded as 1) or absence (coded as 0) of polymorphic bands in the electropherograms, and produce a binary matrix and the GENES program version 2008 (Cruz 2001). The genetic relationships among strains were evaluated using a matrix of genetic distances using the complement of the Jaccard similarity coefficient (CSJ), that does not consider negative similarities and the absence of the product (Cruz and Carneiro 2006). From estimates of the dissimilarities, the lines were grouped using hierarchical UPGMA method (Unweighted Pair-Group Mean Average) with the test of bootstrap (1000 times) to evaluate the consistency of the group (Efron and Tibshirani 1993).

## Results and discussion

In this study we have examined the genetic variation among isolates of *B. thuringiensis*. fAFLP markers generated excellent results in characterizing molecular diversity and genetic relationship within the species of tropical isolates of *B. thuringiensis*. A total of 495 scorable fragments were generated, ranging from 50 to 500 bp, in the 65 *B. thuringiensis* strains when 5 primer combinations were used. Out of 495 fragments 483 were found to be polymorphic, and only 12 fragments were monomorphic (Table 2), and 7 of these fragments were generated by the primer combination *EcoR1*-G + *Mse1*-CC. Our results showed that the *Eco*RI primer with selective nucleotide *Mse1*-C and *Mse1*-CG generated the greatest number of fragments, 130 and 113, respectively. However, *Eco*RI primer with selective nucleotide AC and C with selective nucleotide *Mse1*-CG generated fewer fragments, 77 and 79, respectively.

**Table 2:** **AFLP primer combinations used for the fingerprinting of Bt serovars strains.**

Primer combination	Number of fragments	Number of polymorphic fragments
* *EcoR1*- AC + *Mse1* - CG	77	74
* *EcoR1*-G + *Mse1*- CC	96	89
* *EcoR1*-C + *Mse1* = G	130	129
* *EcoR1*-0 + *Mse1*-CG	113	112
* *EcoR1*-C + *Mse1*-CG	79	79

*AFLP primer combinations.

**Figure 1:**
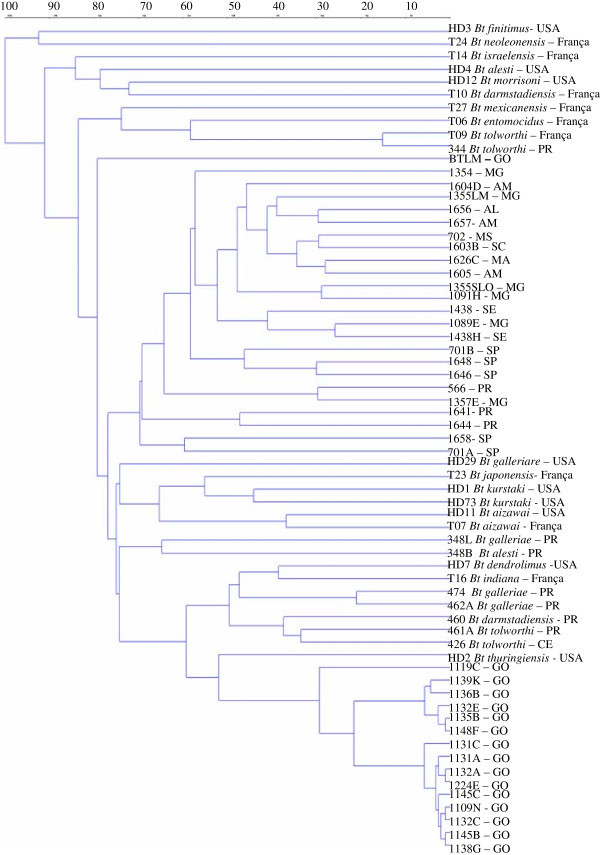
**Dendrogram of genetic similarity of** ***Bacilllus thuringiensis*** **serovars based on AFLP analysis using unweighted pair group arithmetic mean (UPGMA) program for 5 primer combination.**

Genetic distances were obtained using the complement of the Jaccard similarity coefficient (CSJ), and the grouping using hierarchical UPGMA method. Genomic AFLP markers were analyzed through UPGMA cluster analysis to determine the genetic relationship among *B. thuringiensis* isolates (Figure 1). Out of 26 subspecies, strains 344 and T09 (*B. thuringiensis* subsp. *tolworthi*) showed the highest similarity (15%), while isolates HD3 *B. thuringiensis* subsp *finitimus* and T24 *B. thuringiensis* subsp *neoleonensis* were the most genetically distant (92%). *B. thuringiensis* isolates with no subspecies identification, found in samples from Goiás State showed higher similarity forming a group with an average distance of 6%, and the closest subspecies to this group was *B. thuringiensis* subsp *thuringiensis* (HD2) with 52% of similarity. Groups with high similarity were formed with *B. thruingiensis* strains collected in the same State, and other groups composed with *B. thruingiensis* strains collected in different Brazilian regions. No polymorphism was found as a reliable marker among Bt strains efficient and not efficient against fall armyworm.

Among the 65 *B. thuringiensis* strains analyzed, 15 formed a single major cluster, and coincidently these strains were sampled in the same Brazilian State, however more than 200 km apart. The phylogenetic analysis of these Brazilian soil isolates (Figure 1) illustrates the great genetic diversity among strains in the same serovar. However, *B. anthracis* in a previous analysis of a global collection of 78 strains showed little variation among the isolates.

Kumar et al. 1996 found that the low level of similarity between different *B. thuringiensis* subspecies or strains used in his research implied in high levels of genetic diversity which was reflected by the diversity in flagellar H-antigen agglutination reactions and presence of different toxins with different insect specificities. According to Aronson et al. 1986, several factors may contribute to the genetic diversity of *B. thuringiensis* strains, such as the presence of many different plasmids in size and numbers in each strain, and transfer of plasmids via cell mating. Fagundes et al. 2011; found that the number and size of plasmids varied between different strains in the same serovar, however the isolates from Goías State showed similar plasmid profiles, and these informations suggest that these isolates harbor common genes. Carlson, et al. , found that the number and size of plasmids varied between different strains in the same serovar, however the isolates from Goías State showed similar plasmid profiles, and these informations suggest that these isolates harbor common genes. Carlson, et al. found that the number and size of plasmids varied between different strains in the same serovar, however the isolates from Goías State showed similar plasmid profiles, and these informations suggest that these isolates harbor common genes. Carlson, et al. 1996 also reported that the presence or absence of one or more large plasmids and they may be linear or circular during analysis. *B. anthracis* harbors two plasmids and plasmids of similar size have been reported in *B. cereus* and *B. thuringiensis*. These observations support the idea that horizontal gene transfer of plasmid and/or other extrachromosomal markers is an important factor in defining the phenotypes of type I bacillus *B. cereus*-like isolates evolved along apparent large evolutionary distances to give rise to clusters that in more recent times acquired plasmids that conferred insecticidal or other pathogenic phenotypes (Hill et al. 2004). These observations of genetic diversity were also reflected in our results. Lereclus et al. 1984 mentioned that the transposon-like inverted repeats flanking the endotoxin genes are also a factor of genetic diversity.

Few studies have been published using the fAFLP technique to characterize *B. thuringiensis* isolates, but we concluded that AFLP is a highly reproducible, easy to use and relatively fast method which can be applied to different organisms, which can be used for genetic diversity studies, and seems particularly well suited for screening a large numbers of isolates. Moreover, the same protocol can be used in screening at the subspecies level, and demonstrated the value of AFLP technique for the study of diversity between strains of *B. thuringiensis*.
